# Optimism for the UN Proclamation of the Decade of Action on Nutrition: An African Perspective

**DOI:** 10.9745/GHSP-D-16-00117

**Published:** 2016-06-20

**Authors:** Richmond Aryeetey

**Affiliations:** a University of Ghana School of Public Health, Accra, Ghana

On Friday, April 1, 2016, the United Nations (UN) General Assembly proclaimed 2016 to 2025 as a decade of action on nutrition.^1^ Writing as an African living and working in sub-Saharan Africa, where up to 11% of gross domestic product (GDP) is lost to malnutrition^2^ and where malnutrition is declining rather too slowly for anyone’s liking, this proclamation really captured my attention. Another reason this is significant for me and, I reckon, for many others is that this year also marks the beginning of the Sustainable Development Goals (SDGs),^3^ which articulate achieving zero hunger and the even-more aspirational dream of ending all forms of malnutrition by the year 2030. Later this year in August in Rio de Janeiro, Brazil, the Nutrition for Growth Summit will also bring more attention to efforts to eliminate malnutrition around the world. Considering these and other events congregating around such a short duration, it would appear to an outside observer that the world is set for a momentous delivery of something really big for nutrition.

Excited as I am about how all the chips are falling in place, a key question remains on my mind: Do we have the key ingredients to deliver the goods, come 2025 or 2030? My mind goes back to 2008 when the *Lancet* published the first series on maternal and child nutrition, which subsequently triggered the formation of the Scaling Up Nutrition (SUN) Movement.^4^^,^^5^ SUN has become a collective global movement harnessing capacities across governments, civil society, UN agencies, donors, business, and researchers to improve nutrition in countries with the highest burden and vulnerability to malnutrition.^6^

Six years ago, when the SUN Movement was established, many of us in the nutrition community were caught up in the fever of the moment. We became excitedly optimistic that we were finally going to make a major dent in the malnutrition situation in the developing world, and especially in the so-called “high-burden” countries. Although there are signs of significant progress being made through the movement, the “2015 Global Nutrition Report” points to a rather slow rate of change as well as persistant gaps, including insufficient financing and suboptimal country-level capacity needed to address malnutrition at sufficient scale.^7^^,^^8^ But we now have only a couple more years to commemorate a decade of action for the SUN Movement. And in sub-Saharan Africa in particular, we still have a long way to go.

The UN General Assembly proclamation clearly identifies the framework within which the malnutrition situation could be addressed in the next 10 years. However, in the particular case of Africa, I would like to share a few thoughts on what I consider to be missing in the soup, based on past experience. First is that Africans must be at the forefront of the efforts to address malnutrition in Africa. There is a lot of investment already in nutrition in Africa. What is missing is leadership by Africans to make the investment work for the African situation. Much more progress would have been achieved already if African governments had sufficiently prioritized nutrition and made available sustained and adequate financial commitment from their treasuries to address malnutrition. This is especially needed when too many of the countries that are off-course in meeting global nutrition targets are in Africa. Such commitments should be married to a strong process of ensuring accountability for achievement of realistic but sufficiently ambitious targets to address malnutrition in all its forms. Further, African nutrition scientists must take leadership of the evidence economy and generate policy options for decision makers. As the saying goes, “It is never too late to do the right thing.” The time has come to stop having more of the usual situation in which the Global North is driving the efforts to address malnutrition, with Africans playing catch-up.

Africans must be at the forefront of efforts to address malnutrition in Africa.

Second, we have spent much time on repeating research to understand the determinants and characteristics of malnutrition; in some cases, the research agenda is driven from outside Africa and not designed to answer questions relevant to the African.^9^^,^^10^ A key gap waiting to be filled is for African scientists to use systematic approaches to generate policy options for decision making. A decade is too short to do more of the same: analysis and review of the nutrition situation. The available resources will be best used in implementation of existing, proven nutrition-specific and nutrition-sensitive technologies, including biofortification of commonly consumed staples,^11^ promoting dietary diversity, multiple micronutrient supplementation, and scaling up social safety net programs for the most vulnerable in society.^5^^,^^12^ Despite our fears, we should not shy away from the risk of failing. We can learn from our failures. But even more importantly, we can still touch the lives of those who do not show statistically significant improvement.

Third, our governments must be pushed, through grassroots civic movements such as the country SUN civil society networks and traditional and religious leadership, to invest more in nutrition than they are currently doing. The projected economic growth patterns in resource-poor countries will be depleted quickly without governments investing to consolidate the gains that could be made.

Governments must invest more in nutrition.

Finally, more effort should be invested in promoting collaboration across various stakeholders within the African region. Cross-country experience and best-practice sharing can enhance faster progress and reduce costs of program implementation. Within countries, more collaboration across agencies and sectors (government, donors, civil society, research, and the UN) is warranted.

Collaboration across countries can enhance faster progress and reduce program implementation costs.

## Notes

**Peer Reviewed**

## References

[1] United Nations (UN) [Internet]. New York: UN; c2014 Calling attention to chronic hunger, General Assembly decides 2016-2025 will be decade of action on nutrition; 2016 Apr 1 [cited 2016 May 1] Available from: http://www.un.org/press/en/2016/ga11770.doc.htm

[2] International Food Policy Research Institute (IFPRI). Global nutrition report 2014: actions and accountability to accelerate the world’s progress on nutrition. Washington (DC): IFPRI; 2014 Available from: https://www.ifpri.org/publication/global-nutrition-report-2014-actions-and-accountability-accelerate-worlds-progress 10.3945/an.115.008599 PMC4424785 25979494

[3] Sustainable Development Goals: 17 Goals to Transform Our World [Internet]. New York: United Nations; c2016 [cited 2016 May 1] Available from: http://www.un.org/sustainabledevelopment/

[4] Black RE Allen LH Bhutta ZA Caulfield LE de Onis M Ezzati M; Maternal and Child Undernutrition Study Group. Maternal and child undernutrition: global and regional exposures and health consequences. Lancet. 2008;371(9608): 243–260. 10.1016/S0140-6736(07)61690-0. 18207566

[5] Bhutta ZA Ahmed T Black RE Cousens S Dewey K Giugliani E; Maternal and Child Undernutrition Study Group. What works? Interventions for maternal and child undernutrition and survival. Lancet. 2008;371(9610):417–440. 10.1016/S0140-6736(07)61693-6. 18206226

[6] Scaling Up Nutrition [Internet]. Geneva: SUN Movement Secretariat; c2015 The vision of the SUN Movement is a world without hunger and malnutrition; [cited 2016 May 1] Available from: http://scalingupnutrition.org/about

[7] International Food Policy Research Institute (IFPRI). Global nutrition report 2015: actions and accountability to advance nutrition and sustainable development. Washington (DC): IFPRI; 2015 Available from: http://globalnutritionreport.org/the-report/ 10.3945/an.115.008599 PMC4424785 25979494

[8] Fanzo JC Graziose MM Kraemer K Gillespie S Johnston JL de Pee S Educating and training a workforce for nutrition in a post-2015 world. Adv Nutr. 2015;6(6):639–647. 10.3945/an.115.010041. 26567189 PMC4642431

[9] Lachat C Roberfroid D Van den Broeck L Van den Briel N Nago E Kruger A A decade of nutrition research in Africa: assessment of the evidence base and academic collaboration. Public Health Nutr. 2015;18(10):1890–1897. 10.1017/S1368980014002146. 25287557 PMC10271562

[10] Van Royen K Lachat C Holdsworth M Smit K Kinabo J Roberfroid D How can the operating environment for nutrition research be improved in sub-Saharan Africa? The views of African researchers. PLoS One. 2013;8(6):e66355. 10.1371/journal.pone.0066355. 23776663 PMC3680459

[11] Gurmu F Hussein S Laing M. The potential of orange-fleshed sweet potato to prevent vitamin A deficiency in Africa. Int J Vitam Nutr Res. 2014;84(1-2):65–78. 10.1024/0300-9831/a000194. 25835237

[12] Bhutta ZA Das JK Rizvi A Gaffey MF Walker N Horton S; Lancet Nutrition Interventions Review Group; Maternal and Child Nutrition Study Group. Evidence-based interventions for improvement of maternal and child nutrition: what can be done and at what cost? Lancet. 2013;382(9890):452–477. 10.1016/S0140-6736(13)60996-4. 23746776

